# Cervicofacial necrotizing fasciitis after topical application of herbal medicine

**DOI:** 10.1093/jscr/rjab481

**Published:** 2021-11-29

**Authors:** Fernando Miguel Almaguer Acevedo, Barbara Yordanis Hernandez Cervantes, Gabriel Victor Doe Ketemepi, Duniesky Martinez Lopez

**Affiliations:** Department of Surgery, School of Medicine, University of Health and Allied Sciences, Ho, Volta Region, Ghana; Department of Surgery, School of Medicine, University of Health and Allied Sciences, Ho, Volta Region, Ghana; Department of Surgery, School of Medicine, University of Health and Allied Sciences, Ho, Volta Region, Ghana; Department of Internal Medicine, School of Medicine, University of Health and Allied Sciences, Ho, Volta Region, Ghana

## Abstract

Cervical necrotizing fasciitis represents an aggressive form of deep neck space infection with a high mortality rate. The origin is generally odontogenic, in most cases, resulting from a dental abscess. A series of three cases developed after local application of herbal medicine in patients with no co-morbidities and with a history of a toothache in the lower quadrants for >2 weeks is presented. All patients were managed with antibiotics, extraction of offending tooth and serial wound debridement. Two patients recovered with a resultant skin defect on the neck and one died due to multiple organ failure. This case series demonstrated that early diagnosis, prompt surgical intervention and appropriate medical treatment are very important to increase patient survival. Late report to hospital because of financial constraints and over-reliance on herbal preparation could lead to the development of serious complication in patients with dental infections and could even lead to death of the patient.

## INTRODUCTION

Necrotizing fasciitis is an acute, rapidly progressive and potentially fatal soft tissue infection, characterized by extensive tissue necrosis and gas formation in the subcutaneous tissue, fascia and deep tissues. The infection spreads along the fascial planes and can extend into surrounding vessels, nerves and muscles [1, 2]. Cervical necrotizing fasciitis (CNF) represents an aggressive form of deep neck space infection and is associated with a high-mortality rate of 40–76% [3]. The most common cause is odontogenic infection; the original source of the infection is usually an undiagnosed or poorly managed dental abscess. Alternatively, CNF has been documented to result from periodontal disease, tooth impaction or failed tooth extraction [4, 5]. Development of CNF from a topical herbal application and its clinical implication has not been documented in medical literature. A series of three cases secondary to local application of herbal medicine is reported.

## CASE SERIES

### Case 1

A 40-year-old female patient presented to emergency with a history of toothache in lower right quadrant for the past 2 weeks and anterior neck swelling and ulceration for the past 5 days. The patient was seen previously at a dental facility and was advised to extract the offending tooth, but she declined; she rather resorted to topical application of herbal preparations (unknown composition) which led to the rupturing of the swelling with progressively worsening condition when she decided to seek medical attention. She complained of pain, absolute dysphagia and weight loss.

**Figure 1:**
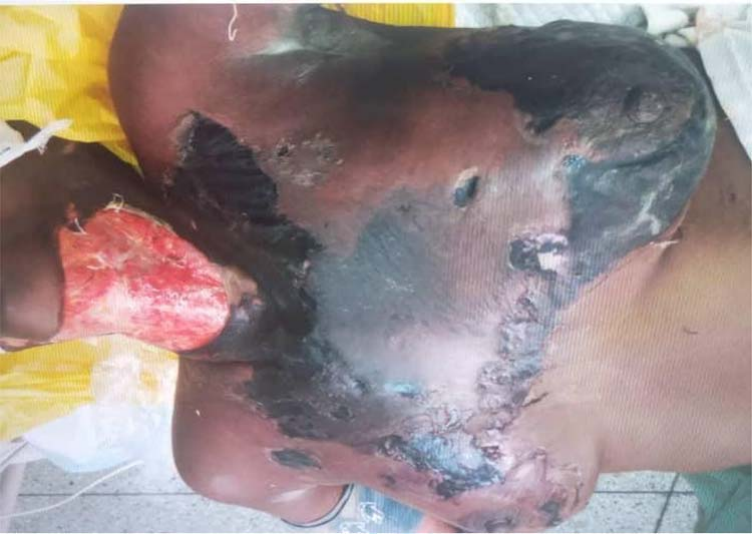
Anterior aspect of the neck and chest.

**Figure 2:**
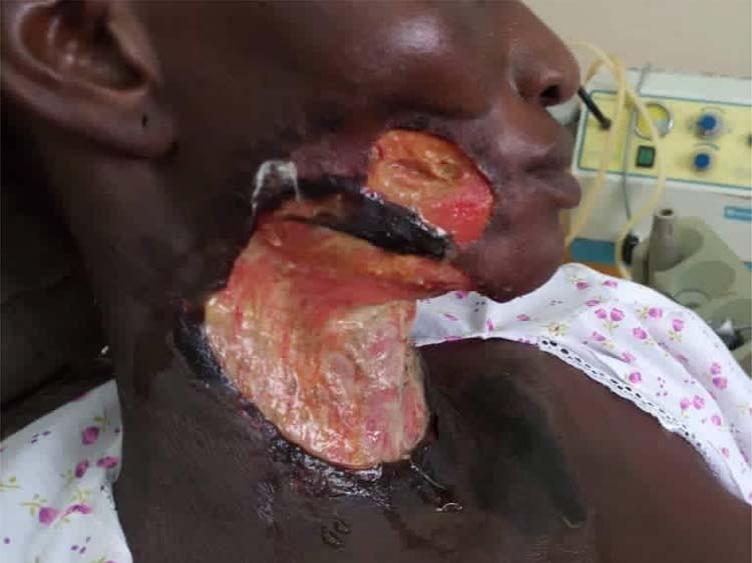
Right lateral facial view.

On physical examination, she was chronically ill-looking and lethargic, icteric, pale and febrile (38.2°C); foul-smelling necrotic tissue was noticed at the anterior and right lateral neck regions. Areas of blistering hyperpigmented patches were present on the anterior chest wall, with the largest extending across the sternum and upper inner quadrants of both breasts from the anterior neck. The right breast was of normal consistency with blistering of the periareolar region. The left breast had hyperpigmented regions appearing like eschar in the upper inner and both lower quadrants (Fig. 1). On the right cheek, there was ulceration communicating with the oral cavity and it measured ~1.5 cm in diametre (Fig. 2). Presence of herbal ointment *in situ* was confirmed. Intraoral examination revealed limited mouth opening, less than one finger breadth, with poor oral hygiene and pus discharge from the lower right molars. Laboratory investigation revealed anaemia, leukocytosis with neutropenia and azotaemia.

**Figure 3:**
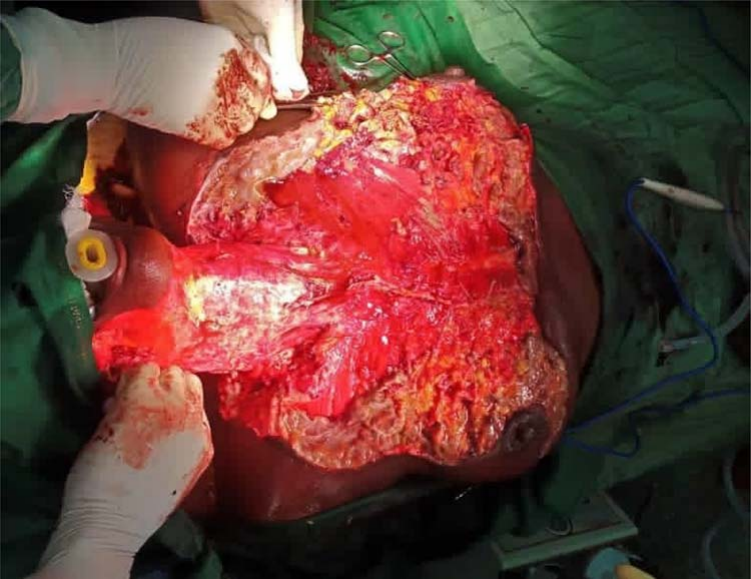
Chest debridement under general anaesthesia.

A diagnosis of CNF with extensions into the breast (mastitis) bilaterally was arrived at. Proper resuscitation was done, including one unit of whole blood and two units of red packed cell, IV triple antibiotic therapy was prescribed and extraction of the offending teeth and surgical debridement under general anaesthesia were done (Fig. 3).

Post-operatively, she was managed at the intensive care unit (ICU) with alternate day dressings, ventilation and ionotropic support; after 8 days, she passed away due to multiple organ failure.

### Case 2

A 68-year-old female patient was admitted to the Accident and Emergency Department with bilateral swelling of the lower jaw. There was a 3-week history of toothache in the lower left quadrant. She resorted to application of topical herbal medications (unknown composition) which led to rapid submandibular swelling, headache and dysphagia to solid foods. There was associated dizziness and general body weakness.

**Figure 4:**
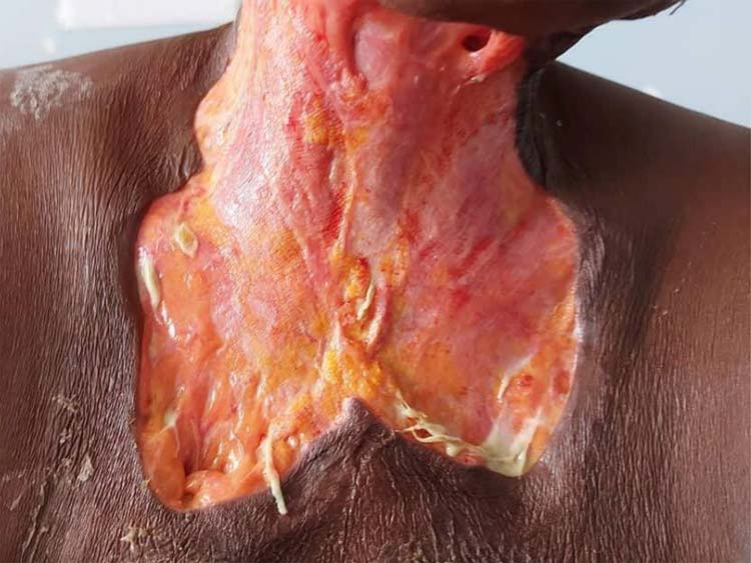
Ulcerations of the neck to the upper third of the chest.

**Figure 5:**
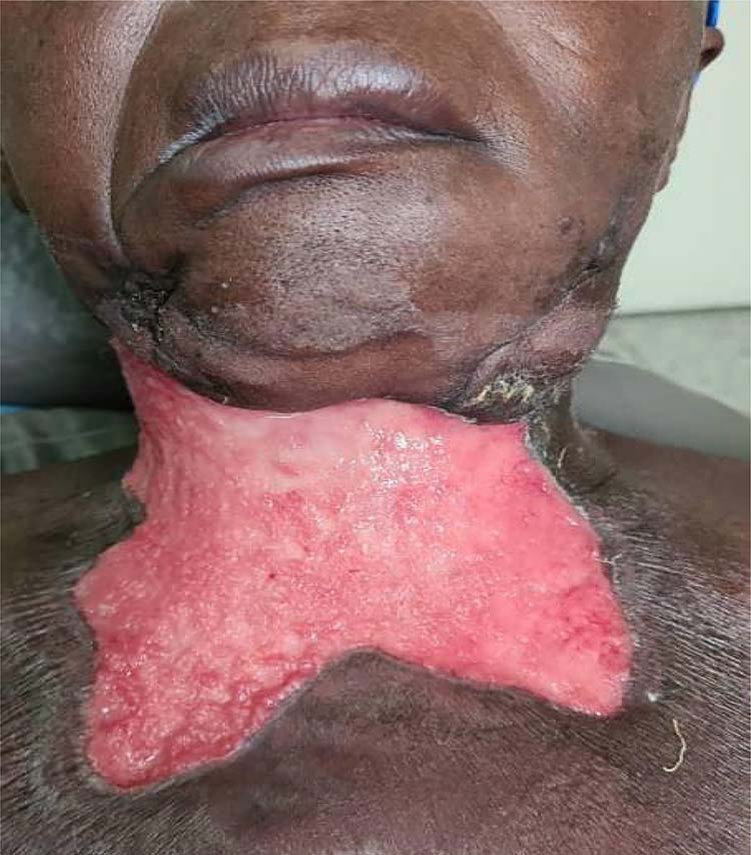
Deviation of the lower lip to the right.

On general examination, the patient was febrile, tachycardic, hypotensive and tachypneic. Extra-oral examination showed ulcerations of anterior neck and lateral cervical regions which extended from the lower border of the mandible to the upper third of the chest (Fig. 4). There was active pus discharge from the wound site with necrotic material at the wound edges. Deviation of the lower lip to the right was also noted (Fig. 5). Intraoral examination disclosed reduced mouth opening, about one finger breadth, with a deep carious cavity on 36 and with retained roots of 48. A full blood count showed anaemia and leukocytosis with neutrophilia. Mildly elevated creatinine was also reported. Orthopantomogram (OPG) confirmed the retained roots of 48 and a deep carious cavity on 36. An impression of CNF secondary to odontogenic infection and complicated by septic shock was formed. The patient was started on intravenous infusions of antibiotics, analgesics and fluids. Patient was haemotransfused with one unit of whole blood and was offered supportive treatment. Wound debridement with two anterior chest incisions under local anaesthesia and extraction of offending teeth with drainage of ~40 ml of frank pus was done. On the sixth day of admission, patient developed bipedal pitting oedema up to ankle, although general condition was improving and it was appropriately managed. The patient was discharged after 17 days on admission for periodic reviews until the wound healed by secondary intention.

### Case 3

A 60-year-old male patient was admitted at the Accident and Emergency Department with lower jaw swelling and ulceration. The patient had episodes of toothache on the lower right quadrant for 2 weeks and then resorted to application of herbal preparation (unknown composition) in order to relief the pain. There were an acute exacerbation of the symptoms and a rapid lower jaw swelling, which ulcerated with purulent discharge that occurred in 3 days before, when he decided to report to the hospital.

**Figure 6:**
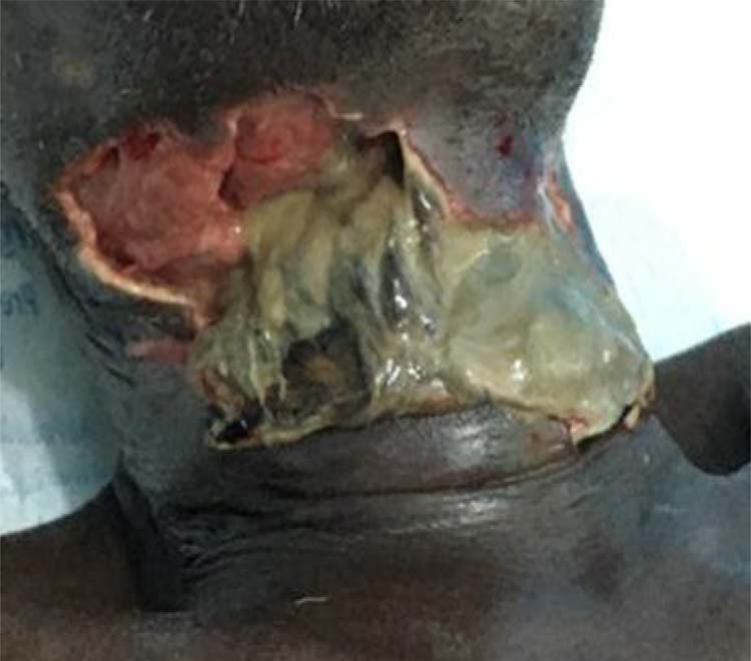
Large ulceration of right submandibular region.

**Figure 7:**
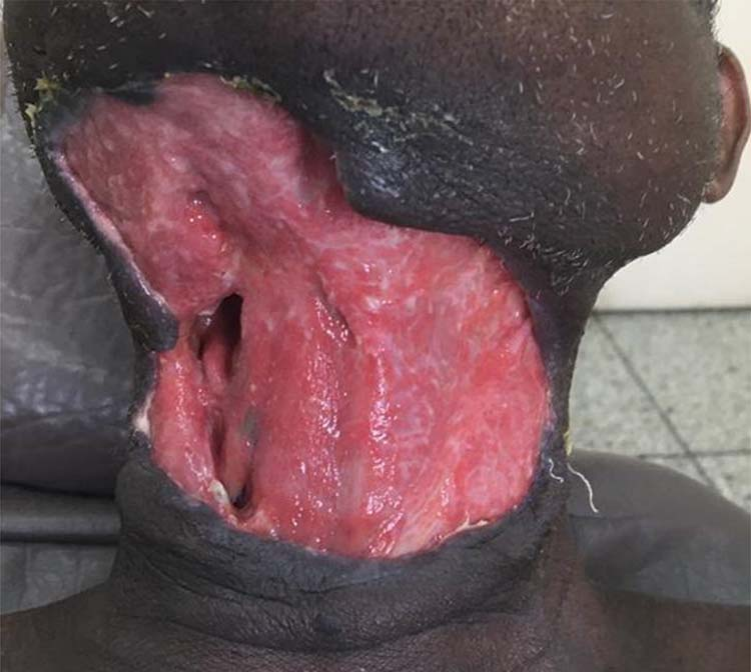
Skin defect after wound debridement.

On examination, the patient was febrile and exhibited signs of mild dehydration, tachycardic and tachypnoeic. There was a large ulceration of the right submandibular region and the entire anterior neck. The floor of the ulcer was covered with slough, and copious discharge of pus from the edges was noted (Fig. 6). Intraoral examination revealed poor oral hygiene, generalized plaques and calculus and tooth number 48 was tender to percussion. Laboratory investigations showed leukocytosis with neutrophilia. OPG showed apical radiolucency of tooth number 48. Diagnosis of CNF was made. IV fluids, antibiotics and analgesics were administered. Wound debridement was done under local anaesthesia with subsequent sessions of daily dressings (Fig. 7). The offending tooth was extracted. The patient was discharged home after 9 days on admission when anterior neck wound was clean. Periodically, follow-ups and reviews were done until healing by secondary intention was achieved.

## DISCUSSION

While odontogenic infections are common, their progression to life-threatening necrotizing fasciitis is relatively rare. The disease may not be recognized until it has progressed significantly [6]. The reason for this is that the onset may be accompanied by nonspecific symptoms of illness [7]. In the three cases reported, the diagnosis was made after at least 2 weeks of patients noticing the first symptoms due to their delay in reporting to the health facility. During that period of time, they tried to cure their toothaches with herbal preparations and sought help from the local herbalists and traditional healers. Studies from sub-Saharan Africa have shown that patients with this condition tend to report late to the hospital probably because of financial constraints and over-reliance on herbal preparations and self-medication [8].

Infection of lower molars was the main aetiology in the three cases. The roots of these teeth are deep to the level of insertion of the mylohyoid muscle along the lingual surface of the mandible. If untreated, infection that originates from these teeth can easily extend to the submandibular space, and subsequently disseminated into surrounding spaces, including sublingual, submental and parapharyngeal spaces [9].

Predisposing factors that have been identified to be associated with CNF include extremes of age, diabetes mellitus, cancer, alcoholism, vascular insufficiencies, organ transplants, human immunodeficiency virus infection, malnutrition, tobacco smoking, arteriosclerosis, chronic renal failure and malnutrition [1, 4, 5, 10]. Investigations and the past medical history of these patients did not reveal any condition which could have predisposed them; they were apparently healthy with no co-morbidities.

CNF is caused by polymicrobial infection, with both facultative aerobic and anaerobic bacteria being implicated [11]. Samples were taken for culture and sensitivity from the three cases, but there was no bacterial growth. The commonest reason for the absence of bacterial growth is that whatever bacteria the swab harvested from the wound surfaces were already dead. Dead or living bacteria look similar, so it will be difficult to tell from the broth if the bacterial cells from the source were still alive or not [12]. A more likely reason in our situation is the lack of ideal medium and growth conditions for the strains involved. It is a known fact that most medical laboratories in low- and middle-income countries, like ours, often come against several challenges at various levels. These include lack of clean water, lack of stable supply of power, unfavourable climatic conditions, dust, inexperienced and poorly trained human resource and a variable supply of poor quality consumables [13].

Complications, such as airway obstruction, mediastinitis, pleural empyema, large vessel thrombosis and septic shock, may be fatal factors that contribute significantly to morbidity and mortality [14]. Two of the patients developed septic shock, and they were managed appropriately, but unfortunately, one patient died in the ICU due to multiple organ failure.

Treatment involves intravenous antibiotics and repeated surgical debridement. In most cases, multiple episodes of debridement are necessary, potentially resulting in significant soft tissue defects. After all nonviable tissue is excised and the infection is cleared, reconstruction with soft tissue flaps may be necessary, especially in patients with massive defects [15, 16]. The patients were managed with IV polyantibiotic therapy, analgesics, IV fluids, wound debridement and extraction of the offending teeth. The two patients who survived had an extensive skin defect on the anterior neck; the main reason why reconstructive surgery was not performed was the inability of patients to afford the procedure.

In Ghana, herbal medicine is an important component of the health care system of the people. In a research conducted in eight communities in the Southern part of Ghana, it was reported that leaves of plants represented 57% of herbal medicines used. Other plant parts that were used were fruits, barks and whole plants. The use of a combination of various plants parts formed 18% of the herbal medicines. Healers are consulted for herbal medicines for the treatment and management of both common and specialized diseases and ailments. The extent to which the people consult the healers is unknown, and the scientific evaluation of the specific uses of the medicines through pharmacological, toxicological and clinical studies in order to ensure the safety of the people consuming the medicines is yet to be determined in many cases [17]. The safe use of herbal medicine demands that herbal medicine producers/herbalists are formally trained, as some of them have previously learned the profession informally from other herbalists. Also, some herbalists with no formal education have a further challenge in calculating dosages and labelling the locally prepared herbal products [18].

Interestingly, the three patients had toothache initially, and they resorted to applying herbal preparation in the form of ointment in the facial region adjacent to the offending tooth which led to a rapid progression of swelling, ulceration and purulent discharge. None of the patients were able to identify the contents of the ointments used and their characteristics. In Ghana, herbal preparation of unknown composition is used for the treatment and management of diseases and ailments. Two or more herbal medicines can be used for the treatment and management of the diseases, and the herbal preparations were most commonly used for the treatment and management of stroke, fevers and diabetes; dental conditions were not found among them [17]. The herbal ointment use by the three patients initiated and exacerbated the progression of soft tissue infection that led to progressive necrosis. We would like to highlight that our patients had none of the risk factors described earlier. They were apparently healthy adults.

## CONCLUSION

This case series demonstrates the serious consequences of the use of herbal preparations of unknown composition in dental infections and of the delay of patients to report to the hospital. Dental infections require a proper assessment by specialized personnel. These infectious processes should never be underestimated. The use of herbal preparations in dental infection could lead to serious complications and to even the death of some patients. Early diagnosis, prompt surgical intervention and appropriate medical treatment are very important to improve the prognosis and patient survival. Further studies should be carried out to determine the contents and effects of topical herbal applications in patients with dental conditions.

## CONFLICT OF INTEREST STATEMENT

The authors have no conflicts of interest to declare.

## FUNDING

The authors have not received financial support from any institution.
